# Is combining serum interleukin-6 and C-reactive protein a reliable diagnostic tool in periprosthetic joint infections?

**DOI:** 10.1186/s13018-020-01864-7

**Published:** 2020-10-02

**Authors:** Cheng Li, Christina Ojeda Thies, Chi Xu, Andrej Trampuz

**Affiliations:** 1 Charité – Universitätsmedizin Berlin, corporate member of Freie Universität Berlin, Humboldt-Universität zu Berlin, and Berlin Institute of Health, Center for Musculoskeletal Surgery (CMSC), Berlin, Germany; 2 Hospital Universitario 12 de Octubre, Madrid, Spain; 3 Department of Orthopaedic Surgery, General Hospital of People’s Liberation Army, Beijing, People’s Republic of China

**Keywords:** Periprosthetic joint infection, Arthroplasty, Serum, Interleukin-6, C-reactive protein, Diagnosis, Meta-analysis

## Abstract

**Background:**

Because there is no single gold standard method for the diagnosis of periprosthetic joint infection (PJI), the combination of valuable methods to evaluate infection appears to achieve a better diagnostic result. The objective of the present study was to evaluate the diagnostic value of serum interleukin (IL)-6 and C-reactive protein (CRP) for the diagnosis of PJI.

**Methods:**

PubMed, Embase, and the Web of Science databases were searched for articles describing PJI diagnosis using serum IL-6 and CRP published between January 1990 and December 2019.

**Results:**

Eight studies were included in the meta-analysis. The pooled sensitivity was 0.84 (95% confidence interval [CI], 0.80−0.88) for the combined method (serum IL-6 and CRP) in series and parallel approaches, 0.87 (95% CI, 0.82−0.90) for IL-6, and 0.84 (95% CI, 0.79−0.88) for CRP. The pooled specificity was 0.85 (95% CI, 0.82−0.88) for the combined method, 0.83 (95% CI, 0.79−0.87) for IL-6, and 0.83 (95% CI, 0.79−0.87) for CRP. The combined method had the highest value for the area under the curve (0.9453), followed by IL-6 (0.9237) and CRP (0.9074). Subgroup analyses showed that the sensitivity of the combined method in parallel tests was higher than that in IL-6 or CRP (94% vs. 89% and 84%, respectively). Serial testing of the combined method showed increased specificity compared to a single indicator (96% vs. 83% and 80%).

**Conclusion:**

The combination of serum IL-6 and CRP was a reliable tool for the diagnosis of periprosthetic hip and knee infection, demonstrating a better diagnostic accuracy than single marker analysis.

## Background

Although a variety of serum, synovial fluid, periprosthetic tissue, and sonication fluid tests are currently performed for the diagnosis of periprosthetic joint infection (PJI), there is no single indicator that can detect infection with 100% accuracy [1]. Combined tests appear to further improve the diagnostic value, although which combined test is more suitable for diagnosis remained unclear [2, 3].

Previous studies have indicated that serum interleukin-6 (IL-6) appears to be a promising tool for the diagnosis of PJI [4, 5]. A meta-analysis of 11 studies reported that the pooled sensitivity and specificity of serum IL-6 were 0.72 (95% confidence interval [CI], 0.63−0.80) and 0.89 (95% CI, 0.77−0.95), respectively [6]. The combined diagnostic method of serum IL-6 and C-reactive protein (CRP) has been used during recent years, and the International Consensus Meeting (ICM) on PJI also place emphasis on its use [7]. IL-6 and CRP have been demonstrated to provide an excellent combined screening test to identify PJI [4, 8]. However, due to the series or parallel tests used in various studies [9, 10], it remains unknown whether these assays are of diagnostic value compared to single parameter analysis for the diagnosis of PJI. Therefore, the objective of the present meta-analysis was to compare the diagnostic performance of IL-6 and CRP with single use parameter detection for the diagnosis of PJI.

## Materials and methods

The Preferred Reporting Items for Systematic Reviews and Meta-Analyses statement was used to guide our methods for the present research [11].

### Search strategy

A search of the PubMed, Embase, and Web of Science databases was performed for studies that assessed the diagnostic value of the combination of IL-6 and CRP for the diagnosis of PJI between January 1990 and December 2019. The following terms refer to the previous publication and search the following medical subject headings (MeSH) or keywords [12]: “arthroplasty or joint prosthesis or joint replacement or periprosthetic joint or prosthetic joint”, “infection or infectious or infected”, “interleukin-6 or IL-6”, and “C-reactive protein or CRP”. The reference lists of the included studies and relevant literature on the combination method of CRP and IL-6 were also manually searched to identify potential studies until no additional articles could be found. In addition, we reviewed the included studies from three relevant meta-analyses [6, 13, 14].

The included studies were selected according to the following criteria: (1) combined method of IL-6 and CRP for the diagnosis of PJI, which were performed in parallel or via serial testing. In parallel testing, a positive result for either IL-6 or CRP was considered positive. In series testing, a positive result in both IL-6 and CRP was defined as positive [15]; (2) the diagnosis standard of PJI was identified by ICM, Infectious Diseases Society of America (IDSA), Musculoskeletal Infection Society (MSIS), European Bone and Joint Infection Society (EBJIS) criteria, or the definition including clinical signs of infection, presence of sinus tract, purulence around the prosthesis, histopathological examination, or the result of synovial fluid, periprosthetic tissue samples, or positive sonication fluid [1, 16–19]; and (3) the number of true positive (TP), true negative (TN), false positive (FP), and false negative (FN) values reported or could be calculated by their corresponding sensitivity and specificity in each article [20].

### Data extraction and study quality assessment

Data extraction was completed independently by two reviewers and subsequently rechecked by a third investigator. Data contained the first author, year of publication, country, enrollment period, study design, number of patients, total cases, infected cases, type of prosthetic joint and bacterium, cut-off, diagnostic criteria, potentially influencing elements, antibiotic use, and the sensitivity and specificity of serum IL-6 and CRP.

All identified studies were evaluated according to the Quality Assessment of Diagnostic Accuracy Studies (QUADAS-2) guidelines by two authors [21]. Any disagreement in the evaluation of the studies was adjudicated by a third author.

### Statistical analysis

For the analysis of the diagnostic value of serum IL-6 and CRP, all statistical analyses were executed using the Meta-Disc software (version 1.4, Unit of Clinical Biostatistics team, Madrid, Spain) [22]. The pooled sensitivity, specificity, positive likelihood ratio (PLR), negative likelihood ratio (NLR), and diagnostic odds ratio (DOR) were assessed. The *I*^2^ statistic was calculated to evaluate the heterogeneity of the included articles, with a range of values from 0 to 100%. If heterogeneity existed between studies, the random effects model was used. Subsequently, subgroup analysis was used to estimate factor affecting the diagnostic accuracy of the combined method for PJI detection.

## Results

### Search results

**Fig. 1:**
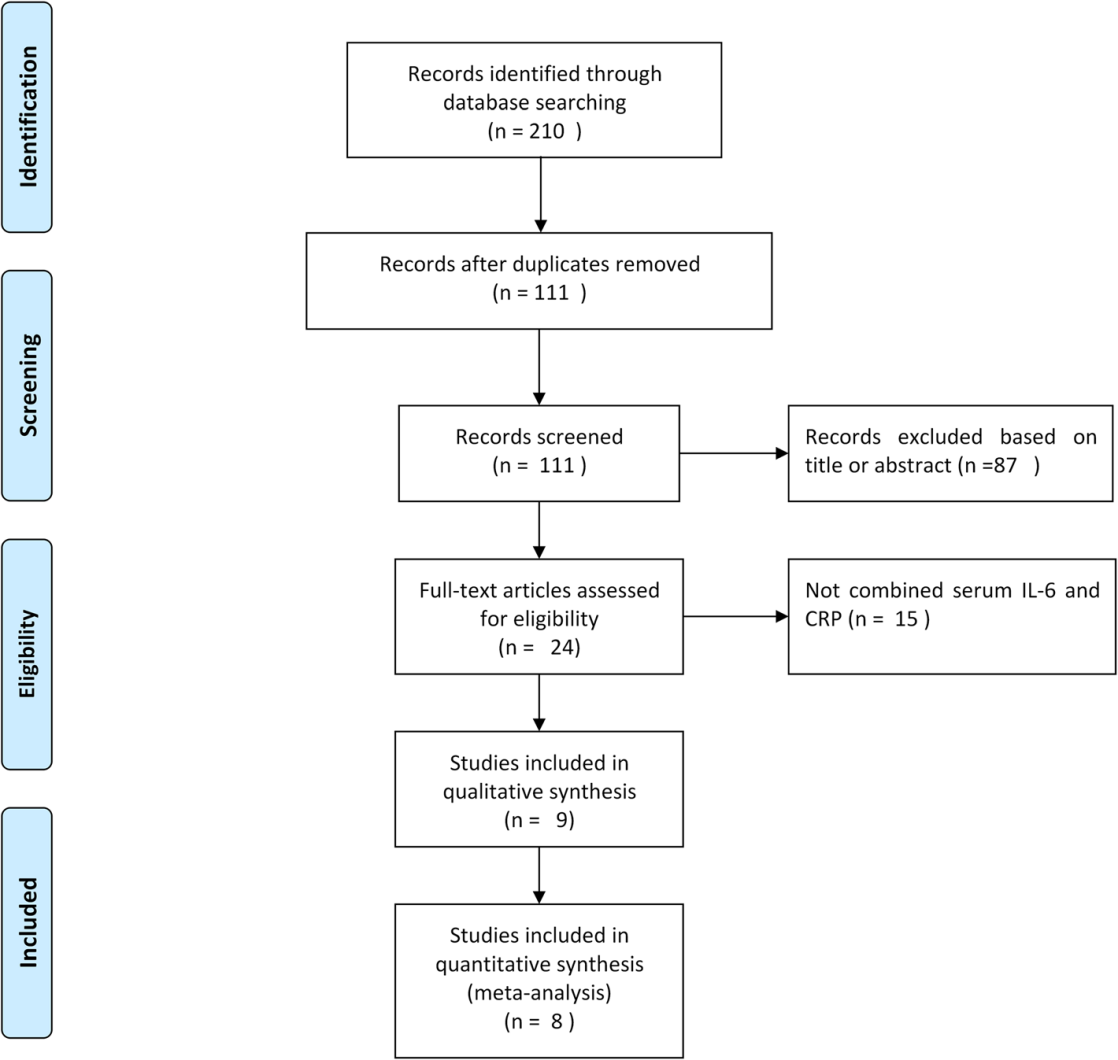
Flow diagram of the selection process for eligible studies

A total of 111 articles were identified following the database search, excluding those excluded due to multiple indexing in different databases. After further review of the title, abstracts, and full articles, eight publications met the inclusion criteria and were included in the analysis (Fig. 1) [4, 8–10, 23–26].

**Fig. 2:**
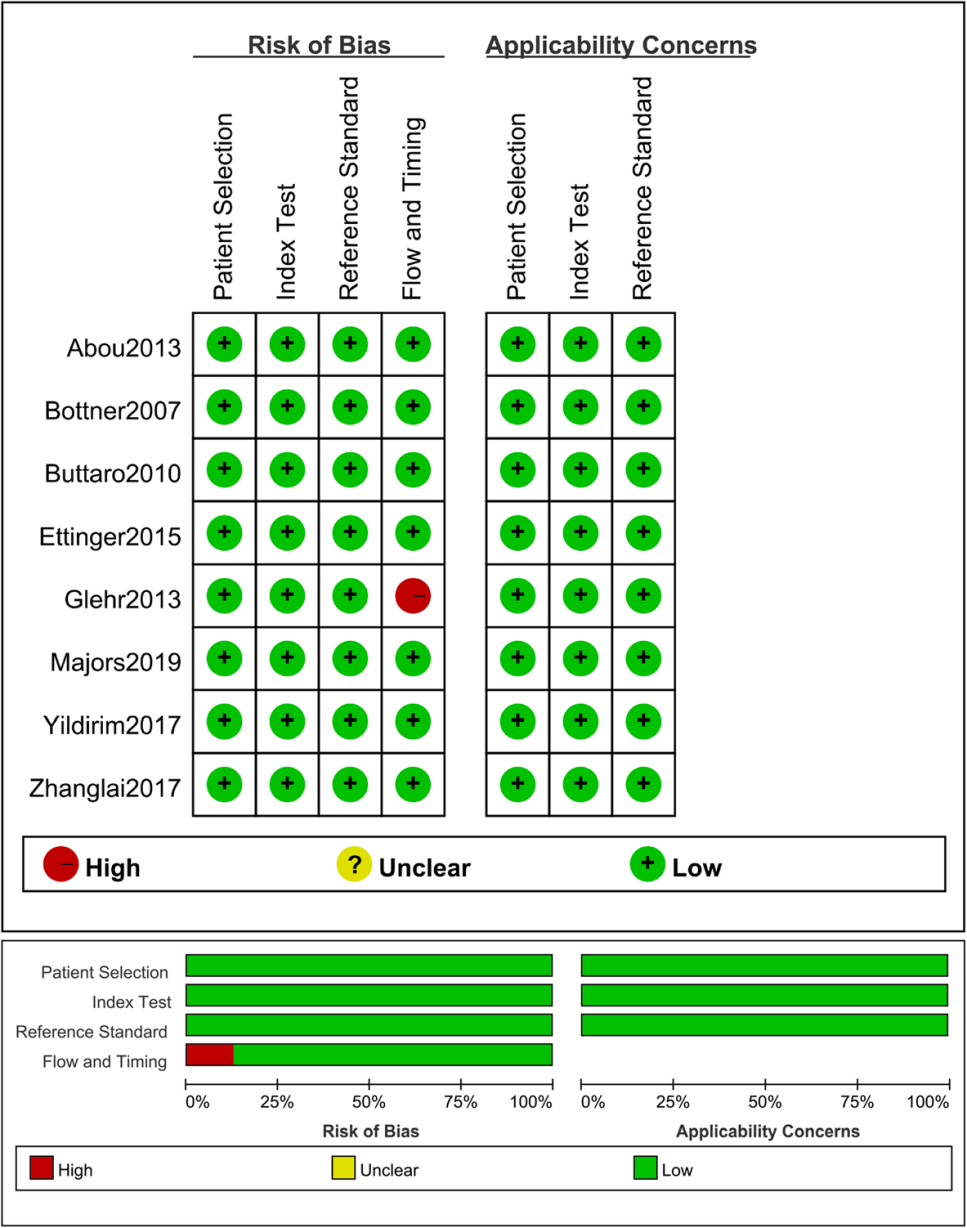
Methodological quality assessment of the included studies

The eight included studies were conducted in seven countries between 2007 and 2019, with 692 total joint arthroplasties (645 patients) identified. Seven articles were written in English and one in Chinese. All studies used an appropriate prospective cohort study design. All studies were related to periprosthetic knee and/or hip infections (Table 1). Seven studies reported bacterial culture results, with staphylococcal infections the most common identified (Table 2). The QUADAS-2 tool was evaluated in all publications and showed that the included studies were of good quality (Fig. 2).

**Table 1:** Characteristics of the included studies for meta-analysis

Reference	Year	Country	Study design	Enrollment period	Patients	Location	Sample	Cut-off	Sen	Spe	Standard	Received antibiotics	Excluded inflammatory disease
[23]	2010	Argentina	Prospective study	February 2007 and July 2008	69	Hip	IL-6	10 pg/ml	36%	94%	H, M	N	Y
							CRP	10 mg/l	72%	91%			
							IL-6 + CRP (Serial tests)		57%	100%			
[24]	2007	Germany	Prospective study	July 2003 and March 2004	78	Hip, knee	IL-6	> 12 pg/ml	95%	87%	H, M	NA	N
							CRP	> 3.2 mg/l	95%	96%			
							IL-6 + CRP (Parallel test)		100%	86%			
[8]	2017	Turkey	Prospective study	April 2010 and December 2012	85	Knee	IL-6	6.6 pg/ml	95%	96%	MSIS	N	Y
							CRP	8.83 mg/l	95%	90%			
							IL-6 + CRP (Parallel test)		99%	98%			
[4]	2013	Egypt	Prospective study	NA	40	Hip, knee	IL-6	> 10.4 pg/ml	100%	90.90%	H, M, P	N	Y
							CRP	> 18 mg/l	100%	86.20%			
							IL-6 + CRP (Serial tests)		100%	99%			
[25]	2019	USA	Prospective study	January 2017 to December 2019	52	Knee	IL-6	> 9.14 pg/ml	81.30%	63%	MSIS	NA	Y
							CRP	> 17 mg/l	66.70%	66.70%			
							IL-6 + CRP (Serial tests)		33.30%	85.0%			
							IL-6 + CRP (Parallel test)		93.8%	45.8%			
[10]	2013	Austria	Prospective study	March 2008 and June 2010	84	Hip, knee	IL-6	4.7 pg/ml	81%	68%	MSIS	Y	Y
							CRP	17.05 mg/l	84%	79%			
							IL-6 + CRP (Parallel test)		84%	68%			
[9]	2015	Germany	Prospective study	NA	77	Hip, knee	IL-6	> 5.12 pg/ml	80%	87.70%	MSIS	N	Y
							CRP	≥ 3 mg/l	80%	64%			
							IL-6 + CRP (Serial tests)		75%	98.20%			
[26]	2017	China	Prospective study	August 2013 and August 2016	160	Hip, knee	IL-6	6.9 pg/ml	96.60%	78%	MSIS	NA	Y
							CRP	8.54 mg/l	79.70%	83.80%			
							IL-6 + CRP (Serial tests)		76.30%	93.10%			
							IL-6 + CRP (Parallel test)		100%	69.3%			

*C* clinical signs of infection, *CRP* C-reactive protein, *H* histological examination, *IL-6* interleukin-6, *M* microbiological or laboratory examination, *MSIS* Musculoskeletal Infection Society, *NA* not available, *N* no, *P* presence of sinus tract or purulence around the prosthesis, *SEN* sensitivity, *SPE* specificity, *Y* yes

**Table 2:** Bacterial species detected in the included studies

Reference	Infected cases	Pathogenic bacteria (numbers)
1	11	Coagulase-negative *Staphylococcus* (4)
		Methicillin-resistant *Staphylococcus aureus* (3)
		*Staphylococcus epidermidis* (2)
		*Staphylococcus viridans* (1)
		*Enterococcus faecalis* (1)
2	21	*Staphylococcus aureus* (7)
		Polymicrobial infection (5)
		*Staphylococcus epidermidis* (3)
		*Enterococcus faecalis* (2)
		*Acinetobacter* (1)
		*Escherichia coli* (1)
		*Streptococcus agalactiae* (1)
		*Streptococcus pyogenes* (1)
3	45	*Staphylococcus aureus* (24)
		Coagulase-negative *Staphylococcus* (9)
		*Staphylococcus epidermidis* (6)
		*Acinetobacter spp.* (3)
		*Enterococcus faecalis* (3)
4	11	*Staphylococcus aureus* (5)
		Coagulase-negative *Staphylococcus* (3)
		*Enterococci* (1)
		*Escherichia coli* (1)
		*Pseudomonas aeruginosa* (1)
5	32	Methicillin-sensitive *Staphylococcus aureus* (9)
		*Streptococcus species* (5)
		Coagulase-negative *Staphylococci* (4)
		*Cutibacterium acnes* (4)
		*Corynebacterium spp.* (3)
		Methicillin-resistant *Staphylococcus aureus* (2)
		*Enterococci* (2)
		*Candida albicans* (2)
		Others (1)
6	55	*Staphylococci* (30)
		Culture-negative infection (16)
		*Streptococci* (8)
		Polymicrobial infection (1)
7	20	*Staphylococcus epidermidis* (8)
		*Cutibacterium acnes* (3)
		Culture-negative infection (3)
		*Staphylococcus capitis* (2)
		*Staphylococcus haemolyticus* (1)
		Polymicrobial infection (1)
		*Staphylococcus auricularis* (1)
		*Micrococcus luteus* (1)

### Diagnostic accuracy of the combined method

**Fig. 3:**
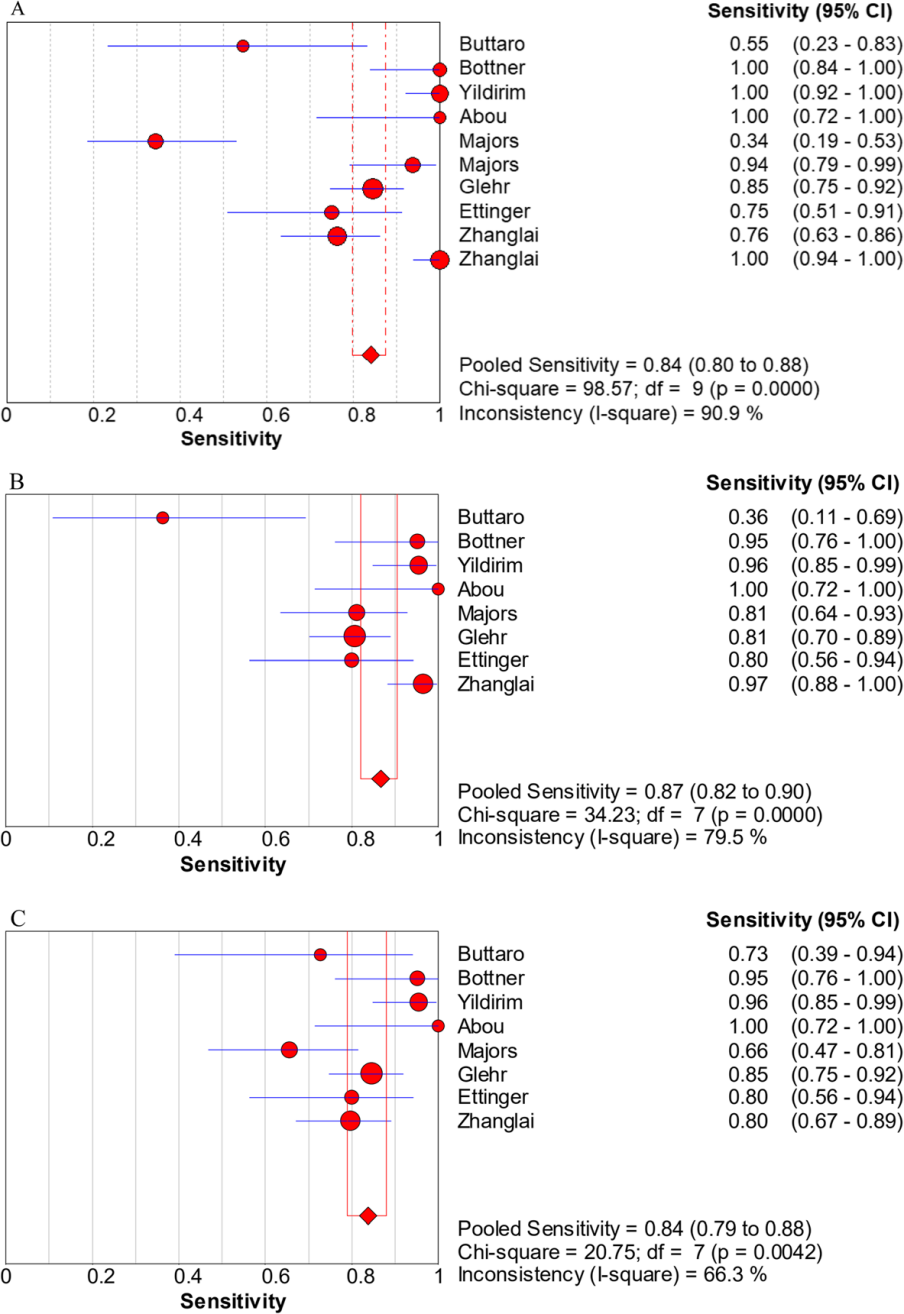
Forest plots of sensitivity for the combined method (**A**), IL-6 (**B**), and CRP (**C**)

**Fig. 4:**
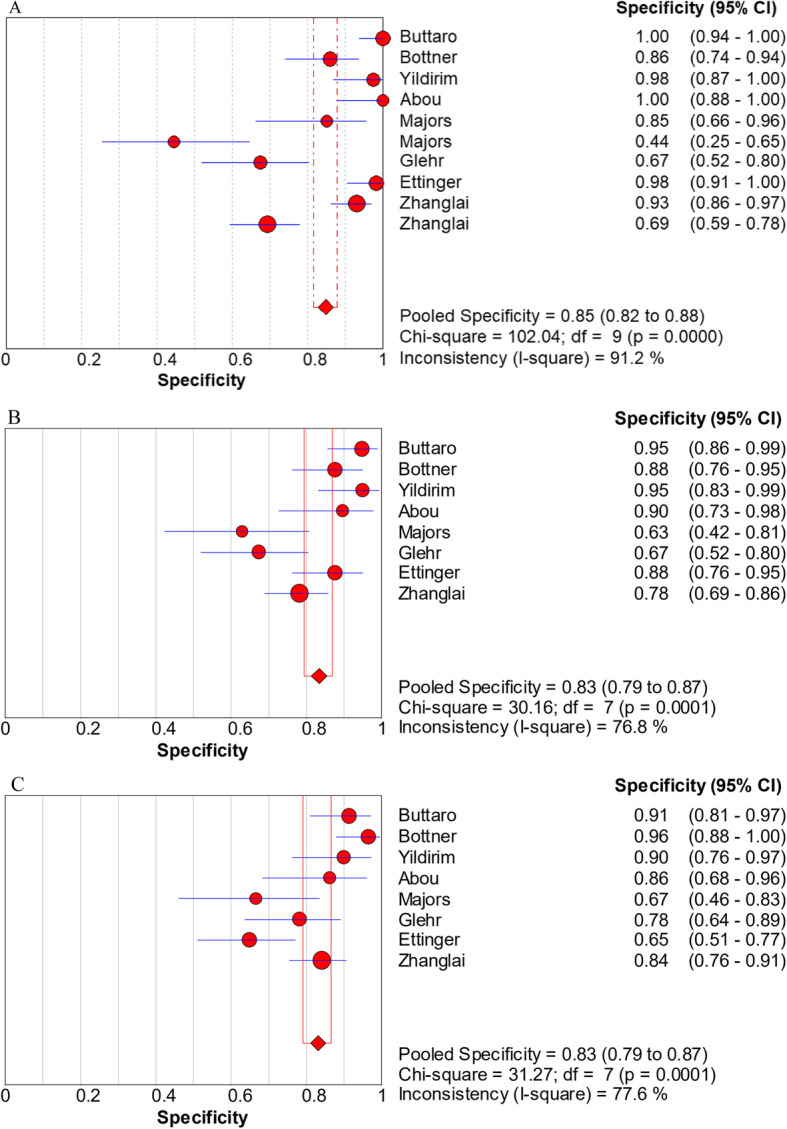
Forest plots of specificity for the combined method (**A**), IL-6 (**B**), and CRP (**C**)

**Fig. 5:**
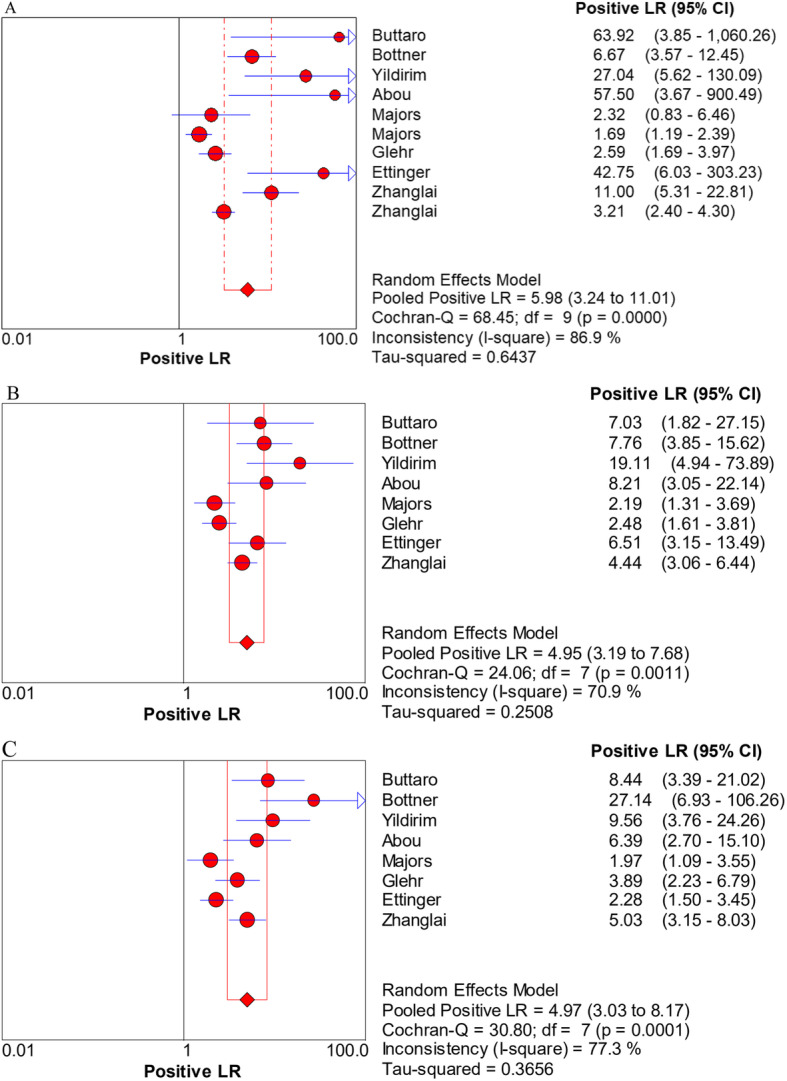
Forest plots of the positive likelihood ratio for the combined method (**A**), IL-6 (**B**), and CRP (**C**)

**Fig. 6:**
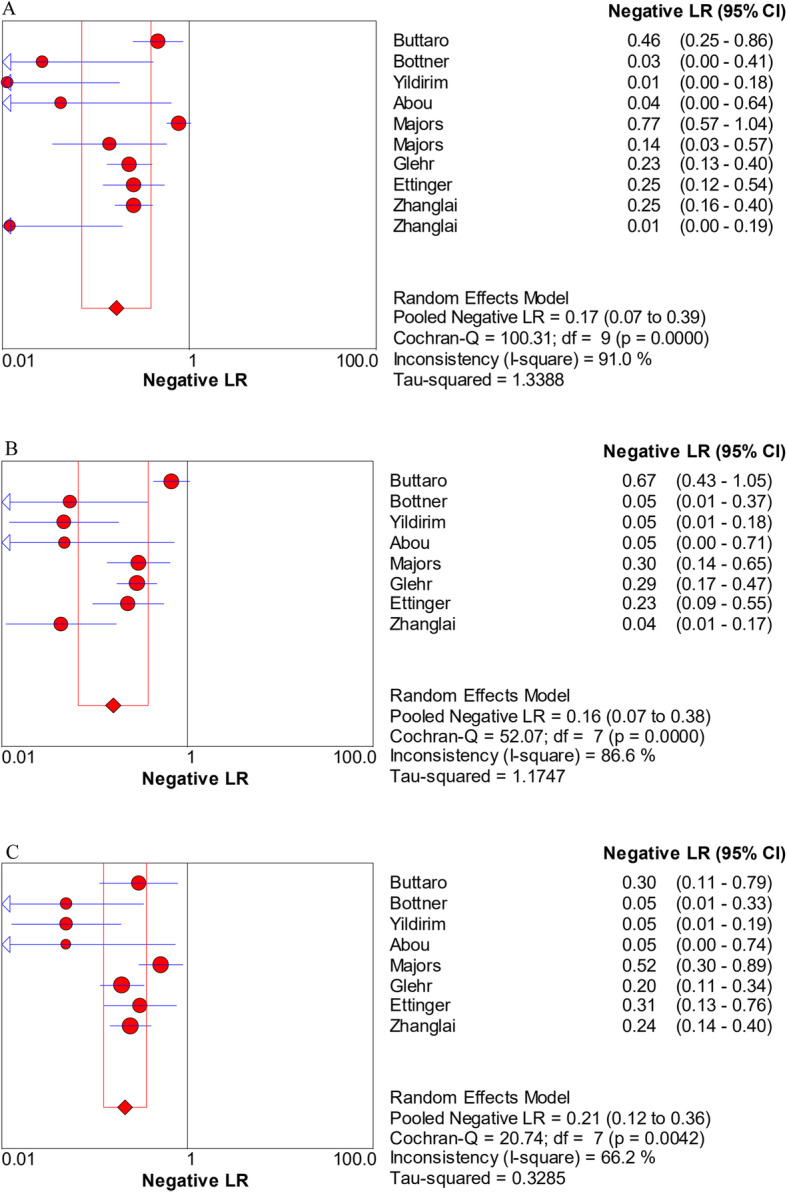
Forest plots of the negative likelihood ratio for the combined method (**A**), IL-6 (**B**), and CRP (**C**)

**Fig. 7:**
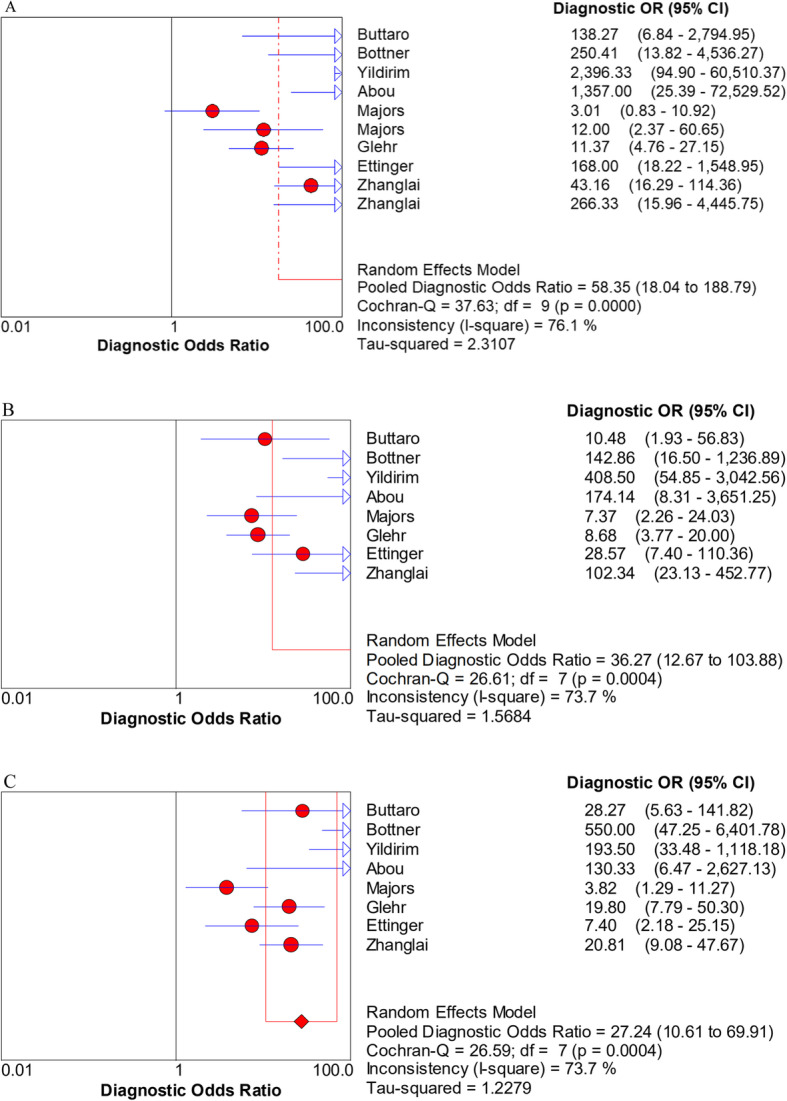
Forest plots of the diagnostic odds ratio for the combined method (**A**), IL-6 (**B**), and CRP (**C**)

**Fig. 8:**
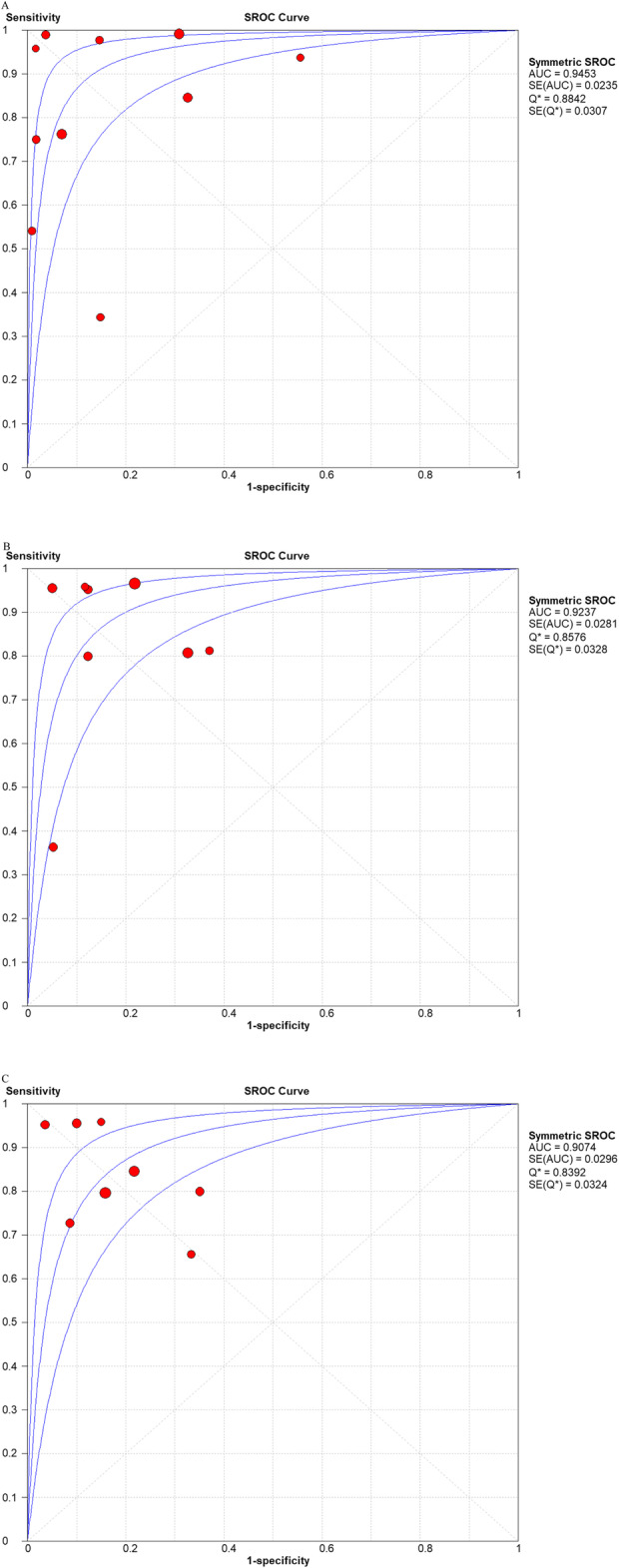
Summary of SROC for the combined method (**A**), IL-6 (**B**), and CRP (**C**)

The pooled sensitivity, specificity, PLR, NLR, and DOR estimates for the detection of PJI using combined serum IL-6 and CRP were 0.84 (95% CI, 0.80−0.88), 0.85 (95% CI, 0.82−0.88), 5.98 (95% CI, 3.24−11.01), 0.17 (95% CI, 0.07−0.39), and 58.35 (95% CI, 18.04−188.79), respectively. The overall pooled sensitivity, specificity, PLR, NLR, and DOR of the IL-6 assay for PJI were 0.87 (95% CI, 0.82−0.90), 0.83 (95% CI, 0.79−0.87), 4.95 (95% CI, 3.19−7.68), 0.16 (95% CI, 0.07−0.38), and 36.27 (95% CI, 12.67−103.88), respectively, while those of serum CRP were 0.84 (95% CI, 0.79−0.88), 0.83 (95% CI, 0.79−0.87), 4.97 (95% CI, 3.03−8.17), 0.21 (95% CI, 0.12−0.36), and 27.24 (95% CI, 10.61−69.91), respectively (Fig. 3, 4, 5, 6, 7). The summary receiver operating characteristic (SROC) plot showed the sensitivity and specificity as well as the 95% confidence intervals and prediction regions, with an area under the curve (AUC) of 0.9453 for the combination of IL-6 and CRP as well as 0.9237 for IL-6 and 0.9074 for CRP (Fig. 8). Significant heterogeneity was found in the combined and single diagnostic tests. Therefore, a random-effects model was used.

### Subgroup analysis

The subgroup results of serum IL-6, CRP, and the combined method are presented in Table 3. Parallel testing of the combined method showed higher sensitivity than IL-6 and CRP (94% vs. 89% and 84%, respectively), while the least specificity 74% vs. 79% and 85%, respectively. In series testing, the combined method demonstrated a lower level of sensitivity than IL-6 and CRP (66% vs. 86% and 77%, respectively), whereas the highest specificity was observed in the combined method with 96%. In the parallel and series tests, the AUC of the combined method was higher than that of single indicators.

**Table 3:** Subgroup analysis of parallel and series tests for PJI diagnosis

Diagnostic method	Number of studies	Sen (95% CI)	Spe (95% CI)	PLR (95% CI)	NLR (95% CI)	DOR (95% CI)	AUC
**IL-6 + CRP (Series and parallel)**	10	0.84 (0.80−0.88)	0.85 (0.82–0.88)	5.98 (3.24−11.01)	0.17 (0.07−0.39)	58.35 (18.04−188.79)	0.9453
IL-6	8	0.87 (0.82−0.90)	0.83 (0.79−0.87)	4.95 (3.19−7.68)	0.16 (0.07−0.38)	36.27 (12.67−103.88)	0.9237
CRP	8	0.84 (0.79−0.88)	0.83 (0.79−0.87)	4.97 (3.03−8.17)	0.21 (0.12−0.36)	27.24 (10.61−69.91)	0.9074
**IL-6 + CRP (Parallel)**	5	0.94 (0.90−0.97)	0.74 (0.69−0.79)	3.67 (2.06−6.53)	0.05 (0.01−0.31)	79.52 (11.18−565.82)	0.9563
IL-6	5	0.89 (0.84−0.93)	0.79 (0.74−0.84)	4.28 (2.46−7.45)	0.12 (0.04−0.33)	43.55 (9.41−201.58)	0.9327
CRP	5	0.84 (0.78−0.88)	0.85 (0.80−0.89)	5.32 (2.76−10.25)	0.19 (0.09−0.39)	32.67 (8.91−119.81)	0.9180
**IL-6 + CRP (Series)**	5	0.66 (0.57−0.74)	0.96 (0.92−0.98)	14.26 (4.05−50.24)	0.34 (0.15−0.76)	53.94 (8.45−344.35)	0.9421
IL-6	5	0.86 (0.79−0.91)	0.83 (0.78−0.88)	4.55 (2.82−7.35)	0.19 (0.06−0.66)	26.49 (8.30−84.55)	0.9056
CRP	5	0.77 (0.69−0.84)	0.80 (0.75−0.85)	3.85 (2.25−6.57)	0.31 (0.20−0.50)	13.28 (5.19−34)	0.8537

*AUC* area under the curve, *CI* confidence interval, *CRP* C-reactive protein, *DOR* diagnostic odds ratio, *IL-6* interleukin-6, *PLR* positive likelihood ratio, *NLR* negative likelihood ratio, *SEN* sensitivity, *SPE* specificity

## Discussion

The present study demonstrates that the combined method of serum IL-6 and CRP can be used for the diagnosis of periprosthetic hip and knee infection. The AUC of the combined method was higher than that of either IL-6 or CRP alone (0.9453 vs. 0.9237 and 0.9074, respectively).

Since there is no gold standard for the diagnosis of PJI, the question of how to accurately judge PJI or aseptic loosening has always been of concern to surgeons, microbiologists, and infectious disease specialists [27]. Traditionally, the combined or single use of the erythrocyte sedimentation rate (ESR) and CRP was most commonly performed in the diagnosis of PJI, which is also one of the criteria of the MSIS definition [17]. One meta-analysis of 12 studies of serum ESR and CRP for the diagnosis of periprosthetic hip infection showed that the sensitivity and specificity were 0.860 (95% CI, 0.825−0.890) and 0.723 (95% CI, 0.704−0.742) as well as 0.869 (95% CI, 0.83−0.899) and 0.786 (95% CI, 0.769−0.803), respectively [28]. However, the diagnostic value of ESR and CRP was not ideal, with a blood test marker for the diagnosis of PJI still required. Serum IL-6 appears to be a superior postoperative inflammatory indicator compared with ESR and CRP. In a patient without complications from hip or knee arthroplasty surgery, the IL-6 level reached its peak value more rapidly than CRP or ESR levels, also rapidly returning to normal [29, 30]. For the diagnosis of PJI, Di Cesare and colleagues found that serum IL-6 had a higher diagnostic accuracy compared with CRP and ESR in diagnosing infection following hip and knee replacement (97% and 83%, 69%, respectively) [5]. A prospective study by Abou et al. [4] also found similar results, namely, that serum IL-6 had better diagnostic accuracy than CRP and ESR (92.5% vs. 87.5% and 82.5%, respectively). Serum IL-6 shows great potential for the diagnosis of periprosthetic hip and knee infection, and IL-6 levels strongly correlated with ESR and CRP using the combined method [25]. The combined test of both parameters has been used recently in the diagnosis of PJI, and some reports have supported that the combination of IL-6 and CRP can improve its diagnostic accuracy [9, 23–25]. The parallel combined screening test could reduce false negative results, increasing the sensitivity and lowering the specificity. Conversely, using series testing could reduce false positives and achieve higher specificity and lower sensitivity [15]. In the present study, eight publications described the use of the combination of IL-6 and CRP in parallel or series tests. Of these, two presented both parallel and series tests and demonstrated a comparison between the combined method of CRP and ESR. Majors et al. [25] showed the sensitivity of IL-6 (> 9.14 pg/ml) and CRP (> 17 mg/l) in a parallel test and specificity in a serial test to be 93.8 and 85%, respectively, whereas the combined CRP and ESR (> 27 mm/h) showed lower sensitivity and specificity (80 and 81%, respectively). Li and colleague [26] reported that the sensitivity of IL-6 (> 6.90 pg/ml) and CRP (> 8.54 mg/l) in the parallel test was higher than that of CRP (> 8.54 mg/l) combined with ESR (> 22.5 mm/h; 100% vs. 86.4%), with a lower specificity to that of CRP and ESR in the serial test (93.1% vs. 96%). In the subgroup of the present study, results were divided into parallel and serial testing. The pooled sensitivity of the parallel test (94%) and the pooled specificity of the serial test (96%) in the combined method was higher than in CRP or IL-6 alone.

All studies included of our meta-analysis were associated with infection after hip or knee arthroplasty. However, the surgical site is likely the potential factor that could affect the diagnostic accuracy. Several reports found that the use of IL-6 or CRP were not suitable for diagnosing infection after shoulder arthroplasty, with the sensitivity of both tests less than 50%. The reason for the low diagnostic value is potentially due to the low virulence of the detected bacterium, such as *Cutibacterium acnes* (*C. acnes*), which commonly occurs in periprosthetic shoulder infections [31, 32]. Majors and colleagues [25] found that the sensitivity and specificity of IL-6 for *C. acnes* in PJI cases was 50 and 59.3%, respectively. Both the sensitivity and specificity were 66.7% for CRP. In coagulase-negative staphylococci infections, the sensitivity and specificity of IL-6 were 100 and 59.3%, respectively, whereas the sensitivity and specificity for CRP was 50 and 66.7%, respectively. The combined method of IL-6 and CRP may improve the diagnostic accuracy of low virulence bacteria. Buttaro et al. demonstrated that the specificity of IL-6 and CRP in serial tests were higher than that of IL-6 or CRP alone (100% vs. 94% and 91%, respectively), with approximately 73% of low virulence organisms presented in the study [22]. Ettinger and co-workers [9] reported a specificity of 98.2% and a sensitivity of 75% for low-grade joint infection. When IL-6 was greater than 5.12 pg/ml and CRP greater than 0.3 mg/dl in serial testing, the specificity of IL-6 or CRP was lower than that of the combined method (87.7% and 64% vs. 98.2%). The author concluded the CRP and IL-6 appears to be the most helpful combination for distinguishing between aseptic loosening and low-grade infection.

In the present study, the different cut-off levels for IL-6 and CRP demonstrated a range from 3 to 18 mg/l for CRP and 4.7 to 12 pg/ml for IL-6. There is no consensus on the use of a single cut-off value, which differ amongst the various studies. For the MSIS definition, the most commonly performed cut-off for CRP was 10 mg/l and 30 mm/h for ESR [17]. However, current thresholds for the diagnosis of PJI should be reconsidered. Bingham et al. [33] reported a sensitivity of CRP with a cut-off of 10 mg/l, which was lower than the 5 mg/l cut-off (85.1% vs. 95.1%). Compared with the threshold of 5 mg/l for CRP and 10 mm/h for ESR, the use of CRP and ESR screening cut-offs of 10 mg/l and 30 mm/h, respectively, would not detect nine PJI cases. The cut-off levels of serum IL-6 for the diagnosis of PJI are still debatable. Previous meta-analysis performed by Xie et al. reported that a serum IL-6 cut-off ≥ 10 pg/ml with greater sensitivity and specificity compared to the cut-off < 10 pg/ml (77% and 98% *vs.* 70% and 80%, respectively), if used a sensitivity and specificity that is greater or equal to 90% as the optimal cut-off from all our included studies. Only two studies meet the criteria in both the serum IL-6 and CRP groups [4, 8, 24], with an IL-6 cut-off of 6.6 or 10.4 pg/ml, and CRP cut-off of 3.2 or 8.83 mg/l potentially achieving a sensitivity and specificity of 90%. When considering studies with a sensitivity and specificity greater or equal to 90% for both IL-6 and CRP, the optimal cut-off is only presented by the study of Yildirim and colleagues [8]. The sensitivity and specificity of IL-6 with a cut-off of 6.6 pg/ml was 95 and 96%, respectively. For CRP, the sensitivity and specificity with a cut-off of 8.83 mg/l was 95 and 90%, respectively. The combined IL-6 and CRP showed a sensitivity of 99% and a specificity of 98%. The high sensitivity and specificity presented in both the individual or combined methods may also be related to the study excluding patients with inflammatory comorbidities, with none of the patients affected by antibiotic treatment. Whether the cut-offs of 6.6 pg/ml for IL-6 and 8.83 mg/l for CRP represent the most optimal cut-offs remains to be explored.

The present meta-analysis has some limitations. First, two of the included studies only had 11 cases of infection [4, 23], and the small sample size potentially affected overall results. Second, although the diagnostic standard was always used to identify cases of infection, the use of different diagnostic standards to estimate the value of diagnostic tools resulted in different sensitivity and specificity values [34]. Third, based on the current publication, two studies showed that the combined method may improve diagnostic results compared to single use in low virulence infections [9, 23]. Due to the relative lack of literature, further research is required to confirm this finding.

## Conclusions

The present meta-analysis supported that the combined serological testing of IL-6 and CRP has a higher diagnostic value than individual testing for diagnosing of periprosthetic hip and knee infection. Further studies are required to confirm the current results.

## Data Availability

Data was extracted from references [4, 8–10, 23–26].

## Abbreviations

AUC: Area under the curve
C: Clinical signs of infection
CI: Confidence interval
CRP: C-reactive protein
DOR: Diagnostic odds ratio
EBJIS: European Bone and Joint Infection Society
ESR: Erythrocyte sedimentation rate
FN: False negative
FP: False positive
H: Histological examination
P: Presence sinus tract or purulence around the prosthesis
IDSA: Infectious Diseases Society of America
ICM: International Consensus Meeting
IL-6: Interleukin-6
M: Microbiological or laboratory examination
MeSH: Medical subject headings
MSIS: Musculoskeletal Infection Society
NLR: Negative likelihood ratio
N: No
NA: Not available
PJI: Periprosthetic joint infection
PLR: Positive likelihood ratio
QUADAS-2: Quality Assessment of Diagnostic Accuracy Studies-2
SROC: Summary receiver operating characteristic
SE: Standard error
Sen: Sensitivity
Spe: Specificity
TN: True negative
TP: True positive
WBC: White blood cell count
Y: Yes

## References

[1] Li C, Renz N, Trampuz A (2018). Management of periprosthetic joint infection. Hip Pelvis.

[2] Tsaras G, Maduka-Ezeh A, Inwards CY, Mabry T, Erwin PJ, Murad MH (2012). Utility of intraoperative frozen section histopathology in the diagnosis of periprosthetic joint infection: a systematic review and meta-analysis. J Bone Joint Surg Am.

[3] Pohlig F, Mühlhofer HML, Lenze U, Lenze FW, Suren C, Harrasser N (2017). Diagnostic accuracy of arthroscopic biopsy in periprosthetic infections of the hip. Eur J Med Res.

[4] Abou El-Khier NT, El Ganainy AE-R, Elgeidy A, Rakha SA (2013). Assessment of interleukin-6 and other inflammatory markers in the diagnosis of Egyptian patients with periprosthetic joint infection. Egypt J Immunol.

[5] Di Cesare PE, Chang E, Preston CF, Liu C-J (2005). Serum interleukin-6 as a marker of periprosthetic infection following total hip and knee arthroplasty. J Bone Joint Surg Am.

[6] Xie K, Dai K, Qu X, Yan M (2017). Serum and synovial fluid interleukin-6 for the diagnosis of periprosthetic joint infection. Sci Rep.

[7] What serum test(s) have the best diagnostic accuracy for periprosthetic joint infection (PJI)? Does the combination of any number of tests increase the diagnostic accuracy? ICM Philly. 2019. Available from: https://icmphilly.com/questions/what-serum-tests-best-diagnostic-accuracy-periprosthetic-joint-infection-pji-combination-tests-increase-diagnostic-accuracy/. Accessed 21 Sept 2020.

[8] Yildirim K, Misir A, Kizkapan TB, Ozcamdalli M, Duygulu F (2017). Neopterin, interleukin-6, procalcitonin, C-reactive protein and PET-CT staining as markers in infected total knee prosthesis, a retrospective analysis. Acta Orthop Belg.

[9] Ettinger M, Calliess T, Kielstein JT, Sibai J, Brückner T, Lichtinghagen R (2015). Circulating biomarkers for discrimination between aseptic joint failure, low-grade infection, and high-grade septic failure. Clin Infect Dis.

[10] Glehr M, Friesenbichler J, Hofmann G, Bernhardt GA, Zacherl M, Avian A (2013). Novel biomarkers to detect infection in revision hip and knee arthroplasties. Clin Orthop Relat Res.

[11] Liberati A, Altman DG, Tetzlaff J, Mulrow C, Gøtzsche PC, Ioannidis JPA (2009). The PRISMA statement for reporting systematic reviews and meta-analyses of studies that evaluate health care interventions: explanation and elaboration. J Clin Epidemiol.

[12] Li C, Ojeda-Thies C, Xu C, Trampuz A (2020). Meta-analysis in periprosthetic joint infection: a global bibliometric analysis. J Orthop Surg Res.

[13] Yoon J-R, Yang S-H, Shin Y-S (2018). Diagnostic accuracy of interleukin-6 and procalcitonin in patients with periprosthetic joint infection: a systematic review and meta-analysis. Int Orthop.

[14] He P, Li S, Huang S, Wa Q, Xu D (2015). Biomarker screening of periprosthetic joint infection and establishment of diagnostic model. J Comput Theor Nanosci.

[15] Franco F, Di Napoli A. Valutazione in parallelo e in serie di test diagnostici multipli. Giornale di Clinica Nefrologica e Dialisi 2016;28:212–215.

[16] Parvizi J, Zmistowski B, Berbari EF, Bauer TW, Springer BD, Della Valle CJ (2011). New definition for periprosthetic joint infection: from the workgroup of the musculoskeletal infection society. Clin Orthop Relat Res.

[17] Parvizi J, Gehrke T, International Consensus Group on Periprosthetic Joint Infection (2014). Definition of periprosthetic joint infection. J Arthroplast.

[18] Osmon DR, Berbari EF, Berendt AR, Lew D, Zimmerli W, Steckelberg JM (2013). Diagnosis and management of prosthetic joint infection: clinical practice guidelines by the Infectious Diseases Society of America. Clin Infect Dis.

[19] Zimmerli W, Trampuz A, Ochsner PE (2004). Prosthetic-joint infections. N Engl J Med.

[20] Li C, Ojeda-Thies C, Trampuz A (2019). Culture of periprosthetic tissue in blood culture bottles for diagnosing periprosthetic joint infection. BMC Musculoskelet Disord.

[21] Whiting PF, Rutjes AWS, Westwood ME. QUADAS-2: a revised tool for the quality assessment of diagnostic accuracy studies. Ann Intern Med. annals.org; 2011; Available from: https://annals.org/aim/fullarticle/474994. 10.7326/0003-4819-155-8-201110180-00009 22007046

[22] Zamora J, Abraira V, Muriel A, Khan K, Coomarasamy A (2006). Meta-DiSc: a software for meta-analysis of test accuracy data. BMC Med Res Methodol.

[23] Buttaro MA, Tanoira I, Comba F, Piccaluga F (2010). Combining C-reactive protein and interleukin-6 may be useful to detect periprosthetic hip infection. Clin Orthop Relat Res.

[24] Bottner F, Wegner A, Winkelmann W, Becker K, Erren M, Götze C (2007). Interleukin-6, procalcitonin and TNF-alpha: markers of peri-prosthetic infection following total joint replacement. J Bone Joint Surg Br.

[25] Majors I, Jagadale VS (2019). Serum interleukin 6 could be a valuable initial diagnostic tool in prosthetic knee joint infections. Eur J Orthop Surg Traumatol.

[26] Li ZL, Zhang BQ, Wang Q, Chen YF, Li RJ, Ke Y (2017). Application of serum inflammatory factors in periprosthetic joint infection. Chin J Jt Surg Electron Ed.

[27] Li C, Ojeda-Thies C, Renz N, Margaryan D, Perka C, Trampuz A. The global state of clinical research and trends in periprosthetic joint infection: a bibliometric analysis. Int J Infect Dis. 2020; Available from: 10.1016/j.ijid.2020.05.014. 10.1016/j.ijid.2020.05.014 32434084

[28] Huerfano E, Bautista M, Huerfano M, Bonilla G, Llinas A (2017). Screening for infection before revision hip arthroplasty: a meta-analysis of likelihood ratios of erythrocyte sedimentation rate and serum C-reactive protein levels. J Am Acad Orthop Surg.

[29] Wirtz DC, Heller KD, Miltner O, Zilkens KW, Wolff JM (2000). Interleukin-6: a potential inflammatory marker after total joint replacement. Int Orthop.

[30] Bilgen O, Atici T, Durak K, Karaeminoğullari BMS (2001). C-reactive protein values and erythrocyte sedimentation rates after total hip and total knee arthroplasty. J Int Med Res.

[31] Villacis D, Merriman JA, Yalamanchili R, Omid R, Itamura J, Rick Hatch GF 3rd. Serum interleukin-6 as a marker of periprosthetic shoulder infection. J Bone Joint Surg Am 2014;96:41–45. 10.2106/JBJS.L.01634 24382723

[32] Grosso MJ, Frangiamore SJ, Saleh A, Kovac MF, Hayashi R, Ricchetti ET (2014). Poor utility of serum interleukin-6 levels to predict indolent periprosthetic shoulder infections. J Shoulder Elb Surg.

[33] Bingham JS, Hassebrock JD, Christensen AL, Beauchamp CP, Clarke HD, Spangehl MJ (2020). Screening for periprosthetic joint infections with ESR and CRP: the ideal cutoffs. J Arthroplast.

[34] Li C, Renz N, Trampuz A, Ojeda-Thies C. Twenty common errors in the diagnosis and treatment of periprosthetic joint infection. Int OrthopInternet. 2019Available from. 10.1007/s00264-019-04426-7.

